# Collaborations between product development partnerships and the World Health Organization

**DOI:** 10.2471/BLT.22.289154

**Published:** 2023-03-31

**Authors:** Matthias Helble, Tanja Kuchenmüller, John Reeder, Jerome H Kim, Bernard Pécoul, David Reddy, Mel Spigelman, Soumya Swaminathan

**Affiliations:** a Research for Health department, Science Division, World Health Organization, Avenue Appia 20, 1211 Geneva 27, Switzerland.; b International Vaccine Institute, Seoul, Republic of Korea.; c Drugs for Neglected Diseases initiative, Geneva, Switzerland.; d Medicines for Malaria Venture, Geneva, Switzerland.; e TB Alliance, New York, United States of America.; f Science Division, World Health Organization, Switzerland.

Over the last two decades, product development partnerships have proven to be a successful model of public–private partnership. Product development partnerships are not-for-profit research and development organizations, typically funded by public and philanthropic organizations focusing on developing new health products to tackle diseases of poverty such as malaria or neglected tropical diseases. A recent report suggests that about 2.4 billion people have benefitted from more than 60 new health technologies introduced by product development partnerships.^1^ With several hundred additional products in the pipeline, product development partnerships are expected to make an even larger contribution to global health, alleviating poverty and increasing global health security. Product development partnerships are active in all stages of pharmaceutical product development, engaging with academic research institutions and the pharmaceutical industry to undertake research and development. Product development partnerships also take on activities aimed at providing equitable access to new health products, especially for vulnerable populations in low- and middle-income countries. The coronavirus disease 2019 (COVID-19) pandemic has shown that product development partnerships are highly relevant in responding to global health crises, among other reasons because they can quickly identify drug candidates for the treatment of mild-to-moderate COVID-19 as well as future coronaviruses.

The World Health Organization (WHO) has long recognized the essential role of product development partnerships in tackling diseases that have high disease burdens in low- and middle-income countries but are neglected by traditional market dynamics. The collaboration between product development partnerships and WHO has often been ad hoc and disease specific; however, in recent years the collaboration has become more consolidated. Furthermore, the COVID-19 pandemic has brought about important new WHO initiatives relevant for product development partnerships as well as changes to the operations of the latter. Here we discuss these recent developments along the biomedical value chain, and show how they have increased the ability of product development partnerships to develop and deliver much-needed new health technologies.

First, the COVID-19 pandemic has triggered a reassessment of how to reduce the time needed for clinical research and development, thereby introducing new health technologies quicker. As part of these efforts, in 2020 WHO published guidance for research ethics committees for rapid review of research during public health emergencies, which allowed to speed up the research on new medications or vaccines.^2^ Soon after the outbreak of the pandemic, WHO released its initial target product profile for COVID-19 vaccines to help inform the development of these vaccines, and has continuously revised the profile since then.^3^ WHO also introduced a new mechanism to provide technical advice to developers in 2021. Under the Coordinated Scientific Advice^4^ procedure, product developers can approach WHO early in the development process and receive advice on the best way to comply with WHO policy and prequalification data needs and processes. The experience during the COVID-19 pandemic has demonstrated that the time for clinical research and development can be substantially reduced. WHO is committed to ensuring that these achievements continue in non-emergency times to facilitate the work of product developers and to shorten the time-to-patients of new products. However, various challenges remain. Not all product developers and other relevant stakeholders are aware of the new services WHO offers. Furthermore, WHO needs to closely collaborate with product development partnerships to ensure that their insights and experiences in clinical research and development are reflected in the normative work of WHO.

Second, the COVID-19 pandemic has revealed the weak research and development capacities in many low- and middle-income countries, including on certain gaps in the research and development continuum. Over the past years, product development partnerships and WHO have made considerable contributions to strengthen research and development capacity in these countries. Since its inception in 1974, the Special Programme for Research and Training in Tropical Diseases, hosted by WHO, has focused on enhancing the research capacities in low- and middle-income countries by strengthening the capacity of individuals, institutions and societies to produce relevant research evidence. Over the years, several thousands of researchers in low- and middle-income countries have benefitted from in-person or virtual trainings organized by the programme as well as from a global network of researchers. Product development partnerships have a long track record of undertaking research and development in low- and middle-income countries, especially in the area of large clinical trials. Already before the pandemic, the clinical research of product development partnerships had engaged more than 550 sites in more than 80 countries.^1^ However, the needs to strengthen local research and development systems in low- and middle-income countries are immense. Product development partnerships and WHO are therefore currently considering ways to collaborate more efficiently, and promote the establishment and use of innovation platforms for research by and for low- and middle-income countries.

Third, in recent years we have seen increased international scientific collaboration, reflected in newly established cooperation, open science projects and synergies across product development partnerships. The COVID-19 pandemic brought some of those initiatives to the forefront. For example, the Drugs for Neglected Diseases initiative and the Medicines for Malaria Venture decided to work together to make available the Pandemic Response Box,^5^ comprised of 400 druggable molecules that could be tested as possible treatments for COVID-19. Other new collaborations include the support of the Access to COVID-19 Tools Accelerator’s Diagnostics Pillar by the Foundation for Innovative New Diagnostics, as well as the efforts by the International AIDS Vaccine Initiative and the International Vaccine Institute to develop or collaborate in the development of COVID-19 vaccines. The emergence of new research collaborations led by low- and middle-income countries to address their needs is another important new trend. For example, in April 2020 the COVID-19 Clinical Research Coalition^6^ started operations and currently has a membership of more than 900 institutions and individuals from 98 countries (mostly low- and middle-income countries). While these new collaborations and networks need to be sustained beyond the pandemic, they demonstrate that in an interconnected world, international scientific collaboration can be quickly mobilized to tackle diseases of common interest, including diseases of poverty.

Fourth, the COVID-19 pandemic has shown that the time between clinical trials and approval can be substantially shortened. WHO Member States and several donors have committed substantial funding to enhance the regulatory capacity of Member States and thereby speed up the overall approval process. For example, the €1 billion Team Europe initiative of the European Commission on manufacturing and access to vaccines, medicines and health technologies in Africa aims to support efforts already underway to strengthen regulatory mechanisms, among other objectives.^7^ These efforts will take time to yield results, and achievements will most likely only become tangible in the medium to long run. However, this work will eventually considerably shorten the time needed to introduce new health products into the market, which will undoubtedly benefit product development partnerships.^8^

Fifth, in response to the inequitable global access to life-saving health products during the COVID-19 pandemic, substantive efforts by low- and middle-income countries are currently underway to increase their local manufacturing capacity of health technologies. Bilateral donors and multilateral development banks are supporting such initiatives. For example, in December 2020, the European Investment Bank launched a 50 million euro pharmaceutical investment initiative to strengthen local production of active pharmaceutical ingredients in Africa.^9^ Other examples include WHO’s recently established mRNA (messenger ribonucleic acid) technology transfer hub in South Africa as well as a global biomanufacturing training hub in the Republic of Korea, with the aim of training specialists in the latest manufacturing technologies, and to enable the transfer of technology and know-how to manufacturers in low- and middle-income countries.^10^ However, while these initiatives had a promising start, they have shown to be rather costly and, critically, hinge on the continued support of governments and the international donor community. If they can be maintained, the product development partnerships could benefit from the strengthened local manufacturing capacity. Product development partnerships have always been eager to work with pharmaceutical producers located in disease-endemic countries, but often struggled to find local companies capable of producing quality health technologies at affordable prices. Improved local manufacturing capacity in low- and middle-income countries could thus speed up the production, delivery and access to new health technologies.

The COVID-19 pandemic has undoubtedly affected the work of product development partnerships and WHO. However, both actors have been able to respond to the new challenge, making significant contributions to the pandemic response. While product development partnerships quickly shifted their focus to product development and delivery, WHO used its normative mandate to introduce institutional and procedural innovations to accelerate product development. Product development partnerships and WHO have also been successful in strengthening local research and development capacity and building international research networks. With more than 375 new technologies^1^ in the pipelines of product development partnerships and increased manufacturing capacity in low- and middle-income countries, a unique potential exists for enhanced disease control and access to care. However, fulfilling this potential will not be automatic. Product development partnerships and WHO need to increase and institutionalize their collaboration, for example by setting up a WHO–product development partnerships technical working group, to ensure that their resources are leveraged. Finally, sustained commitment by governments of low- and middle-income countries as well as continued support of the international donor community are needed to successfully build on the current momentum and to achieve a long-term shift towards an accelerated, more equitable and sustainable introduction of new health technologies to tackle diseases of poverty.

## References

[1] Keeping the promise: product development partnerships’ role in the new age of health research and product development. Keeping the Promise Report: 2021. Available from: https://www.keepingthepromisereport.org/ [cited 2022 May 8].

[2] Guidance for research ethics committees for rapid review of research during public health emergencies. Geneva: World Health Organization; 2020. Available from: https://www.who.int/publications/i/item/9789240006218 [cited 2022 May 11].

[3] WHO Target Product Profiles for COVID-19 Vaccines. Version 3. Geneva: World Health Organization; 2020. Available from: https://www.who.int/publications/m/item/who-target-product-profiles-for-covid-19-vaccines [cited 2022 May 11].

[4] WHO Coordinated Scientific Advice for health product R&D. Geneva: World Health Organization; 2023. Available from: https://www.who.int/activities/optimizing-research-and-development-processes-for-accelerated-access-to-health-products/who-coordinated-scientific-advice-for-health-product-r-d [cited 2023 Mar 23].

[5] The Pandemic Response Box. Geneva: Drugs for Neglected Diseases initiative; 2023. Available from: https://dndi.org/research-development/portfolio/pandemic-response-box/ [cited 2023 Mar 23].

[6] COVID-19 Clinical Research Coalition [internet]. Geneva: COVID-19 Clinical Research Coalition; 2023. Available from: https://covid19crc.org [cited 2023 Mar 23].

[7] €1 billion Team Europe initiative on manufacturing and access to vaccines, medicines and health technologies in Africa. Brussels: European Commission; 2021. Available from: https://ec.europa.eu/commission/presscorner/detail/en/ip_21_2594 [cited 2022 Apr 11].

[8] Another triumph of science, but defeat for access? Geneva: Drugs for Neglected Diseases Initiative; 2021. Available from: https://dndi.org/wp-content/uploads/2021/08/DNDi-COVID-19-Policy-Report-2021.pdf [cited 2022 Apr 10].

[9] EIB launches EUR 50 million Africa pharmaceutical manufacturing initiative. Luxembourg: European Investment Bank; 2020. Available from: https://www.eib.org/en/press/all/2020-377-eib-launches-eur-50-million-africa-pharmaceutical-manufacturing-initiative [cited 2022 Apr 6].

[10] Establishment of a COVID-19 mRNA vaccine technology transfer hub to scale up global manufacturing, Expression of Interest. Geneva: World Health Organization; 2021. Available from: https://www.who.int/news-room/articles-detail/establishment-of-a-covid-19-mrna-vaccine-technology-transfer-hub-to-scale-up-global-manufacturing [cited 2022 Apr 4].

